# Developmental Precursors of Social Brain Networks: The Emergence of Attentional and Cortical Sensitivity to Facial Expressions in 5 to 7 Months Old Infants

**DOI:** 10.1371/journal.pone.0100811

**Published:** 2014-06-26

**Authors:** Santeri Yrttiaho, Linda Forssman, Jussi Kaatiala, Jukka M. Leppänen

**Affiliations:** Tampere Center for Child Health Research, School of Medicine, University of Tampere, Tampere, Finland; Lancaster University, United Kingdom

## Abstract

Biases in attention towards facial cues during infancy may have an important role in the development of social brain networks. The current study used a longitudinal design to examine the stability of infants' attentional biases towards facial expressions and to elucidate how these biases relate to emerging cortical sensitivity to facial expressions. Event-related potential (ERP) and attention disengagement data were acquired in response to the presentation of fearful, happy, neutral, and phase-scrambled face stimuli from the same infants at 5 and 7 months of age. The tendency to disengage from faces was highly consistent across both ages. However, the modulation of this behavior by fearful facial expressions was uncorrelated between 5 and 7 months. In the ERP data, fear-sensitive activity was observed over posterior scalp regions, starting at the latency of the N290 wave. The scalp distribution of this sensitivity to fear in ERPs was dissociable from the topography of face-sensitive modulation within the same latency range. While attentional bias scores were independent of co-registered ERPs, attention bias towards fearful faces at 5 months of age predicted the fear-sensitivity in ERPs at 7 months of age. The current results suggest that the attention bias towards fear could be involved in the developmental tuning of cortical networks for social signals of emotion.

## Introduction

While the social skills of an individual reach their full capacity after years of development, critical phases of this development may occur already during the first year of life. Therefore, the identification of the precursors of social cognition in infants is important. Several studies suggest connections between the key nodes of the emotional and social brain networks [1], especially the amygdala, and cortical face processing circuitry [2], [3]. Thus, studying the emergence of cortical face sensitivity in infants may provide a critical window on the ontogeny of social cognition.

Behavioral observation of infant visual attention points to an extremely early onset of face processing abilities. Already newborn babies prefer faces or face-like stimuli over non-facial objects [4], [5] and discriminate between different facial expressions [6]. By 3 months of age, infants acquire a more specialized preference for smiling happy faces [7], and a categorical discrimination between some facial expressions by the age of 5 months [8]. Rather than a monotonic improvement, the acquisition of face expertise may consist of transient sensitive periods for the development of particular capabilities. Sensitive periods or “experience-expectant” mechanisms [9] could underlie the momentary waning of face preference towards the second month [5]. The preference for smiling faces at 3 months, likewise, changes into bias towards fearful faces by the age of 5 to 7 months [10]–[12]. Such early biases in attention towards facial cues have been suggested to have an important role in canalizing the development of social brain networks [13].

The development of face-selective neural populations in infants has been targeted predominantly by studies using electroencephalography (EEG) and less frequently by other modalities of neuroimaging. The earliest signs of face-sensitive cortical activity in infancy have been observed in positron emission imaging of fusiform face areas (FFA) already at 2 months of age [14] and by event-related potentials (ERPs) from the age of 3 months [15]. The face-selective N170 response [16] generated in adult FFA [17], [18] is developmentally preceded by the N290 and P400 responses [15], [19]. Despite early face-effects in the N290, the correspondence to the adult N170 remains incomplete during the first year of life. For instance, the inversion effect, or ERP-sensitivity to the orientation of face stimuli [20], [21] is not reached until 6 months of age in the P400 [22]. In N290, the effect of face-inversion has been found by the age of 3 months [23], although contrasting results [19] suggesting a delayed onset of this effect, have been reported. Sensitivity of infant ERPs to distinct facial expressions, absent at 3 months in fearful vs. neutral contrast, emerges by the age of 6 months [24]. The effects of facial expression on infant ERP components such as N290, P400, and the Negative central (Nc) have been documented at ages of 7 and 9 months [24]–[30].

The relationship between the N170, as an index of cortical activity related to the structural encoding of faces [16], [31] and the modulation of ERPs by emotional content of facial expressions remains to be clarified. While the effects of facial expressions on ERPs in the N170 latency range have been suggested to indicate emotional modulation of face-specific activity [27], [32], Rellecke et al. [33] have recently argued for the independence between the face-related activity and the coincident emotion-related ERPs in adults. Whether the modularity of face- and emotion-sensitive cortical processes is present already during infancy, or develops later on, has not been established. Interestingly, unlike in the adult N170 [34], the amplitude of the 4-month-old infant's N290 is larger for faces with direct than averted gaze [35]. Thus, the infant N290 may be less specialized to structural encoding than the adult N170. In order to disentangle face- and emotion-related activity underlying face-sensitive ERPs in infants, experimental conditions contrasting both facial (face vs. non-face) and emotional content (emotive vs. neutral facial expressions) are needed.

In the current study, we focused on the connections between the development of behavioral attention preferences towards facial expressions and that of cortical face-sensitive areas. A recent study with a large sample size (N = 73) showed that the attentional bias towards fearful faces is present already at the age of 5 months [12], suggesting that this component of fear bias may emerge earlier than suggested by previous studies with smaller samples [10], [36]. However, no longitudinal studies combining measurements of both behavioral and ERP responses to facial expressions are available from this developmental period. Thus, the stability of the fear-bias and the relationship between the fear-bias in overt attention and cortical activity elicited by facial expressions await further elucidation. Interestingly, such biases in attention towards social stimuli [13] as wells as subcortical face-processing pathways [3] have been suggested to lay developmental foundation for cortical processing of conspecific signals. These views would, thus, predict that attentional biases dictate the development of cortical sensitivity to faces and facial expressions. However, cortical sensitivity to faces and facial expressions might also influence attentional preferences towards these stimuli during early infancy. In order to address these questions, we combined the measurement of infant gaze patterns and electroencephalographic (EEG) data during the presentation of distinct facial expressions as well as non-face control stimuli at 5 and 7 months of age in a longitudinal design. Our aims were 1) to examine the stability of the behavioral fear-bias across 5 and 7 months of age, 2) to identify fear-sensitive cortical activity in ERPs recorded over posterior scalp regions at 5 and 7 months of age, 3) to analyze the relationships between the scalp topographies of fear- and face-sensitive ERPs, and 4) to explore the correlations between the gaze and ERP patterns of fear-sensitivity both within and across measurements. Given recent results for the early onset of the overt attentional bias towards fear at 5 months [12] and the hypothesized role of attention biases in guiding the development of social brain networks [13], we were particularly interested in testing the prediction that early biases in attention towards fear are associated and may also developmentally precede cortical fear-sensitive activity.

## Methods

### 1. Ethics Statement

Ethical permission for the study was obtained from the Ethical Committee of Tampere University Hospital and a written informed consent was given by the parents of the participants before the start of the study.

### 2. Participants

The current study was implemented as part of an ongoing longitudinal study started in April 2012 (Tampere, Finland) with planned follow-ups from the age of 5 months until the age 48 months. The attention disengagement data from this longitudinal study have been reported in previous publications [12], [36], and a sub-sample of the EEG data was used as an example dataset in a methodological study [37]. In the current study, we re-analyzed the attention disengagement data to assess developmental stability and focused on the relations between attention disengagements and ERPs in the context of emotive face stimuli at 5 and 7 months of age. The average ages at measurements were 5.1 months (SD = 0.1, Range: 4.8–5.4 months) and 7.1 months (SD = 0.1, Range: 6.8–8.0 months). A total of 125 (55 females) infants participated in these assessments. The participants were healthy, and largely from urban middle-class families. Premature birth (n = 1), fussiness (n = 2) or technical problems during testing (n = 6), and experimenter's error (n = 1) lead to the rejection of 10 participant from all further analyses. The final sample sizes varied between different analyses according to the specific inclusion criteria as explained below (detailed in ‘5. Gaze acquisition and analysis’, ‘7.2. ERP extraction’, and ‘8. Statistical analyses’).

### 3. Face stimuli

**Figure 1:**
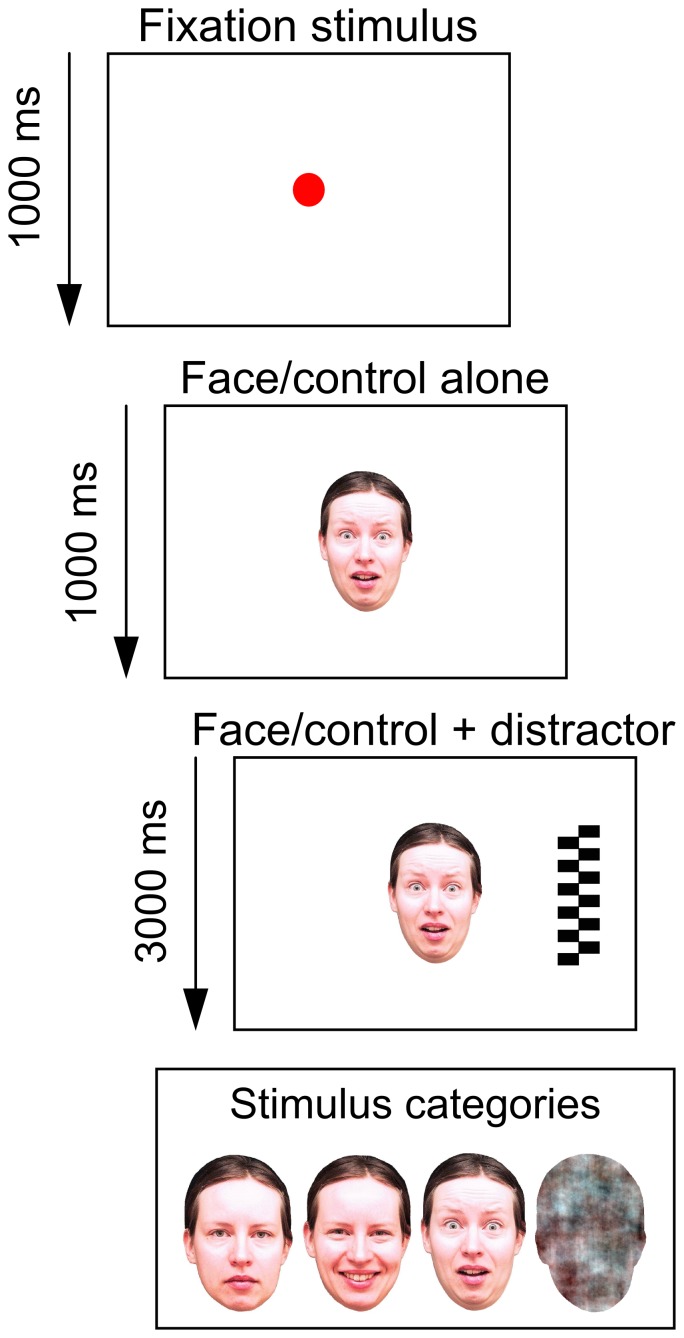
The overlap paradigm. A face or a control stimulus was presented in the center of the screen after the participant fixated on an expanding red circle (fixation stimulus). A distractor was added to the right or to the left of the central stimulus after 1000 ms from face/control onset. The central stimulus was presented until the end of each trial, thus, overlapping in time with the distractor. The sequence of events and stimuli in the paradigm are shown with the duration of each event (top). The stimuli categories presented in the central location (neutral, happy, and fearful faces as well as phase-scrambled control stimuli) are shown in the bottom panel.

The experimental stimuli consisted of both face stimuli and non-face control stimuli. The faces were photographed images of two female models portraying fearful (FE), happy (HA), and neutral (NE) expressions (Figure 1, bottom). A validation of these facial stimuli as good examples of the intended emotional categories has been provided by Peltola et al. [10]. The non-face stimuli were produced by randomizing the phase spectrum of one of the face pictures from each model, thus, controlling for low level visual features (e.g., brightness and amplitude spectrum) of the stimuli between faces and non-face stimuli. Both faces and non-faces were cropped to the outline of face/head and subtended 15.4° and 10.8° (visual angles) on a 23″ monitor. The stimuli were viewed at the distance of about 60 cm in a dimly lit room.

### 4. Attention disengagement paradigm

In order to capture biases in infant attention, we used the Overlap paradigm [38], where a high-contrast or other salient (“pop-out”) stimulus is inserted as a distractor along the initially presented and attended stimulus (for an illustration of the paradigm, see Figure 1). A typical infant response to the presentation of the distractor is a gaze shift towards this novel stimulus. As the gaze shift reflects an active attentional process of disengagement from the initial stimulus, the paradigm can be used to probe differences in attention allocation between distinct stimuli [39]. In the current study, different facial expressions were first presented in the center of the screen for a duration of 4 seconds in each trial. After 1 second from the face onset, a peripheral target appeared 13.6° randomly on the left or the right side of the face (or non-face control) stimulus. The distractor stimuli were black and white checkerboards or circle arrays with a height and width dimension of 15.4° and 4.3°, respectively. Before each trial, an animated attention-grabber (a red circle which dilated periodically from 0.4° to a size of 4.3°) was presented in the screen center. When the infant fixated on the attention-grabber, the experimenter initiated the presentation of the face (or non-face) stimuli. Facial expressions from one of the two models, counter-balanced across participants, were shown in the first 24 trials after which face stimuli from the second model were used. The stimuli were presented in random order with the constraints that each of the four expressions from both models were presented 6 times and the flanker was presented on the same side of the screen no more than four times in a row.

### 5. Gaze acquisition and analysis

Infants' gaze was recorded by digital video camera (Canon ZR960 & QuickTime or iMovie software) and corneal-reflection eye-tracking (Tobii TX300, Tobii Technology, Stockholm, Sweden). Allocation of attention to distinct face stimuli was investigated through the probability of gaze disengagement from the centrally presented face to the peripheral distractor. That is, trials were coded as containing an attention disengagement from the face or a sustained dwell of attention at the face. Valid attention disengagement was defined as a saccade towards the distractor within 150 to 1000 ms after distractor onset. Only trials where the participant fixated (engaged) to the face and maintained gaze within the face for at least 70% of the time preceding gaze disengagement (or the 1000 ms post-stimulus time limit) were coded. Gaze disengagements were extracted from eye-tracking data using custom Matlab code (gazeAnalysisLib) [40].

### 6. EEG acquisition

EEG was recorded simultaneously while running the Overlap paradigm by using a high-density EGI HydroCel 128-electrode net (Electrical Geodesics, Inc.) and was sampled at 250 Hz. During the measurement, the accompanying parent held the baby on her/his lap without touching the electrode net. Electrode impedances were measured at the beginning of each session and saved in separate files. Data were initially (first 33 participants) acquired until a count of 72 trials was reached or until the participant could no longer remain attentive to the stimuli. Because most participants became inattentive or fussy before reaching the last trial, we lowered the maximum number of trials to a level that was acceptable for most infants (48, which equaled to 12 presentations of each stimulus category). Overall, an average of 48.3 (SD = 10.2) and 46.4 (SD = 4.5) trials were presented at 5- and 7-months measurements, respectively.

The inter-stimulus interval (ISI), measured between the onsets of consecutive face/control stimuli, was determined by the disengagement paradigm to always exceed 4 seconds. As the rate of stimulus presentation was contingent on the infant fixating the attention-grabber and the experimenter manually initiating the trial, the average ISIs were 10.4 (SD = 3.0) and 9.3 (SD = 2.2) seconds for the measurements at 5 and 7 months, respectively.

### 7. EEG analysis

#### 7.1. EEG preprocessing

EEG preprocessing was based on a combination of visual quality control of participant compliance and automated artifact detection using the Eegtool software [37] which incorporates key preprocessing functions from the EEGLAB toolbox [38]. First, EEG epochs during which unwanted participant behaviors were identified in synchronized video recordings of the participants were rejected. Such behaviors included gaze shifted away from the central stimulus prior to the onset of the lateral distractor, saccades, blinks, sucking the pacifier, contraction of the oral or other facial muscles, prominent tongue movements, excessive body movements, infant or parent touching the electrodes, parent moving the infant, and infant being outside the angle of view of the video.

The steps in the automated EEG preprocessing were 1) low-pass filtering at 30 Hz, 2) segmentation of the data to epochs spanning −100 ms to 800 ms around stimulus onset, 3) detrending the epoch, 4) rejecting channels with high impedance values (>200 Ω during calibration), and 5) aligning the EEG signal to the 100-ms pre-stimulus baseline. The data in each epoch was then scanned (automatically) for artifactual EEG signal in each channel using a maximum-amplitude-based criterion. Channels in a given epoch indicating absolute potentials greater than 150 µV were marked bad and replaced with data interpolated from acceptable channels using spherical interpolation. However, if the number of bad EEG channels in an epoch was greater than 12 (i.e., about 10% of the 128 electrode channels), the entire epoch was rejected. Finally, the EEG signal was re-referenced to the average from all electrodes.

#### 7.2. ERP extraction

To examine face- and fear-sensitivity in ERPs, we combined epochs from specific stimulus conditions in the following way: Face condition includes fear (FE), happy (HA), and neutral (NE); Non-Face condition includes phase-scrambled control stimulus (CS); Fear condition includes FE; and Non-Fear condition includes HA & NE. ERPs were initially extracted separately from all electrode channels as averages across epochs given that at least 5 acceptable epochs were acquired from the participant/condition. Although this criterion is lower than that typically used in infant ERP studies (i.e., 8–10 trials/condition), the reduced signal-to-noise ratio (or effect size) at the level of individual infants is compensated by the relatively large overall sample size in the current dataset. The average number of epochs included in the ERP analyses for the face-effect was 15.5 and 16.0 at 5 and 7 months, respectively. For the fear-effect the corresponding mean numbers of epochs were 11.2 and 12.0. It is also of note that the within-subject analyses were complemented by group-level bootstrapping analyses (as described below) that used all available data and were not affected by any criteria for trial count or number of available trials per participant. From the measurement at 5 months, 60 and 75 participants were included in the analyses of face- and fear-sensitivity, respectively. At 7 months, acceptable data for the analysis of face-sensitivity was acquired from 99 participants, and 100 participants were included in the analysis of fear-sensitivity. In longitudinal repeated-measures (across 5 and 7 months of age) analyses, only participants with acceptable data from both measurement ages were qualified (N = 50 and N = 63 for the analysis of the fear- and face-effects, respectively).

The conventional within-subjects analysis of ERPs was complemented by group-level analyses using grand-averaged ERPs calculated from all available trials in the dataset. This approach enables statistical analyses of data without excluding participants with low trial counts as well as visualization of variability around the grand average [35]. Using bootstrapping, we calculated resampling distributions of ERP waves from a pool of accepted epoch data from all participants. We further produced confidence intervals (CIs) using the basic bootstrap intervals to allow statistical analyses of differences between stimulus conditions. These CIs were calculated for both ERPs from specific stimulus conditions (e.g., Fear) and for their differences (e.g., Fear vs. Non-Fear). Statistical significance of the difference between two conditions can, thus, be stated when the difference wave, and its confidence limits, take non-zero values. In the current analysis, we sampled epochs from a set of electrode channels with replacement. Thus, the size of the pool of epochs input to bootstrapping analysis depended on the number of channels in the channel set and the number of acceptable epochs (depends on stimulus condition) from all participants. Analyses concerning the potential effects of variations in the number of acceptable epochs between stimulus conditions (e.g., Face vs. Non-Face) are reported in Supplementary material (Supplement S1). The toolboxes [37], [41] used for EEG analyses are open source and custom scripts used for data analyses are available from the authors.

### 8. Statistical analyses

#### 8.1. Behavioral analyses

We summarized the behavioral disengagement data into indices of face- and fear-bias based on the probability of attention shifts, 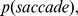 from the face stimulus to the peripheral target (disengagement). The bias scores were calculated as follows:
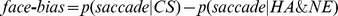


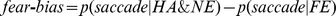



That is, in the calculations of the face-bias, the face condition (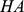 is happy and 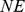 is neutral stimulus) was contrasted to the non-face control condition (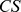 is control stimulus). In calculating the face-bias we sought to minimize the effect of emotional cues on the score variable. In particular, as fearful faces have been shown to suppress attention shifts to peripheral targets [10], [12], [36], [42], the fearful condition was not included in the calculation of the face-bias. The fear-bias was based on the difference between the fearful stimulus condition (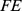) and the average across non-fearful face conditions. The number of accepted participants (with ≥3 trials per condition) for the calculation of the face- and the fear-bias was 74 and 75 for the 5-months visit and 103 and 103 for the 7-months visit. From these participants, an average of 8.2–9.1 trials (SD = 2.4–3.2) and 8.8–9.1 trials (SD = 2.5–2.7) were acquired per condition at 5- and 7-months visits, respectively. In order to assess the reliability of the behavioral scores, we calculated Spearman ρ coefficients between the bias scores obtained from the first and last 24 trials measured during the 5-months visit. These split-half reliabilities for fear- and face-bias scores from the 5-months visit were ρ = .28 (p<.05, N = 49) and ρ = .39 (p<.01, N = 50), respectively. The disengagement probability as such, rather than the bias scores based on their difference, had a split-half reliability of ρ = .64.

A comprehensive analysis of the behavioral data has been presented in our previous article [12]. In the current study, we analyzed the consistency of the disengagement probability and the behavioral bias scores across visits at 5 and 7 months using Sprearman correlation coefficients. We, further, analyzed these correlations in reference to the score reliability.

#### 8.2. ERP analyses

**Figure 2:**
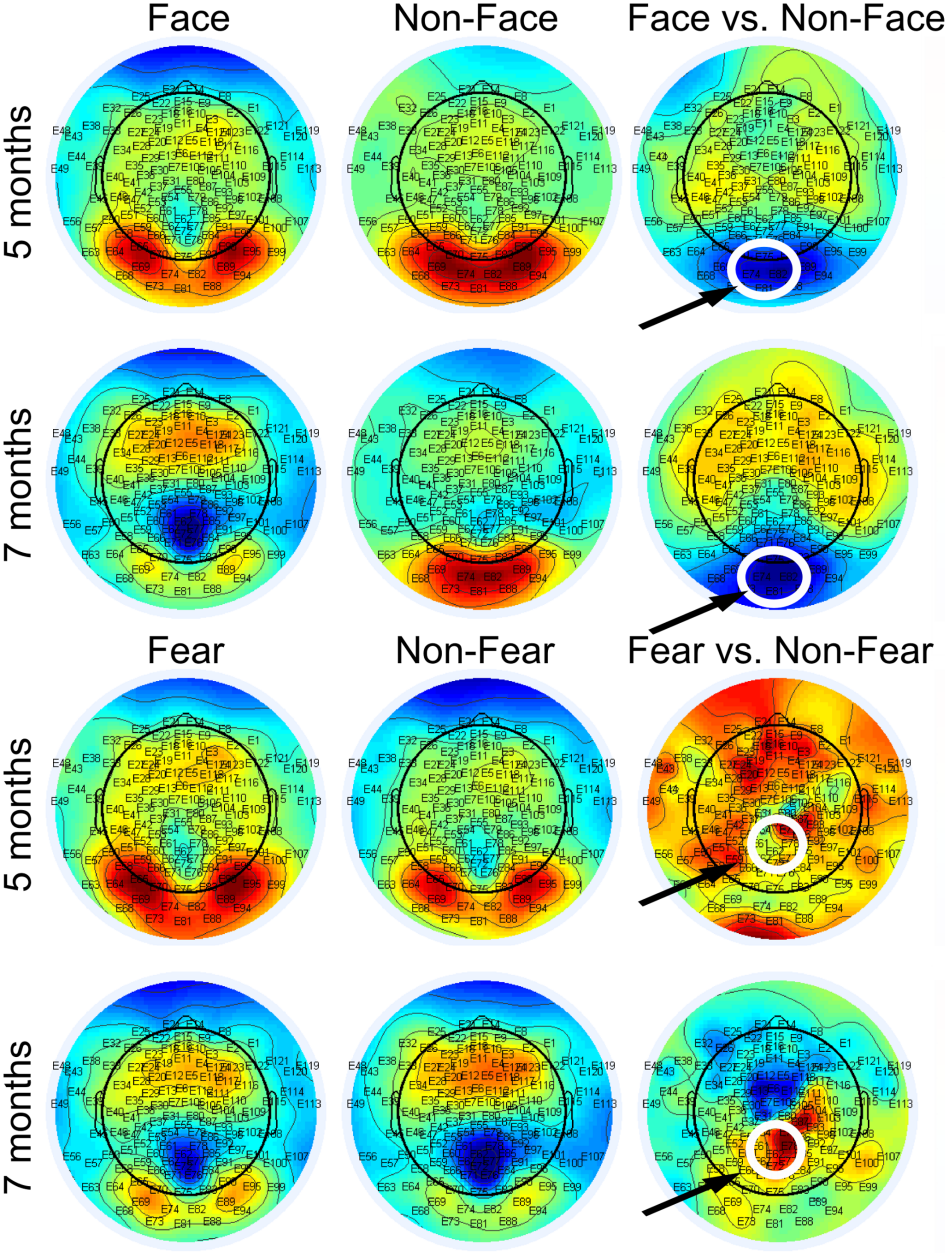
Face- and fear-sensitivity plotted in ERP scalp topographies from the 248–348-ms latency range corresponding to the N290 response. The top-most panels show ERPs elicited by face and non-face control stimuli and their difference in Studentized values from the measurements at 5 and 7 months of age. The bottom-most panels show the corresponding topographies for the fear- and the non-fear conditions as well as from their difference. The largest differences between face- and non-face conditions were found in the occipital electrodes (E70, E73, E74, E75, E81, E82, E83, and E88). For the fearful vs. non-fearful conditions the largest differences were found in the parieto-occipital channels (E72, E62, E67, E61, E54, E77, E78, and E79). Channel groups are encircled (white line) in the scalp maps.

Analyses of the ERP data focused on posterior channels given previous studies showing differences in ERPs to faces vs. non-faces at posterior sites 200–300 ms after stimulus onset [14] as well as larger ERP positivity to fear-related vs. neutral/happy cues, starting at the latency of the N290/P400 components [27], [43] (see also [10], [24], for evidence for differential activity at frontocentral regions). Initial analyses of the current data with similar electrode groupings to those used in a previous high-density study [44] replicated previous findings regarding differential ERPs for faces at 5 and 7 months and fearful facial expressions at 7 months of age (reported in [37], shown in Supplement S1). However, these analyses provided no information about the spatial distribution of face- and fear-effects and their overlap over posterior scalp areas. Thus, in order to determine the electrode sites that were maximally sensitive to different stimulus conditions (Face vs. Non-Face and Fear vs. Non-Fear), we calculated differences between ERPs from pairs of conditions. The analyses focused on the amplitude of the N290 component (i.e., 248–348 ms post-stimulus time) given previous research linking this component with face processing and our own preliminary analyses of the current dataset showing overlap in face- and fear-sensitivity at this latency range (Supplement S1). The differences, for each time-point, were normalized using the t-statistic as follows:
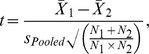
where
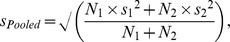
and 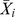 is mean amplitude, 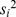 is variance, and 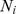 is sample size (number of ERPs). Based on visual inspection of the scalp topographies of these t-scores (Figure 2), we identified clusters of differential activity for face and fearful stimuli at posterior sites.

After identifying clusters of electrodes exhibiting maximal face- and fear-sensitivity over posterior scalp sites the statistical significance of the effects at group level were further tested by using the bootstrapping analyses and all available epochs as described above. Also, the overlap in scalp distributions of the face- and the fear-effects were investigated through Sensitivity × Location interactions on ERP amplitudes. The factor Sensitivity referred to the difference either between the Fear and the Non-Fear stimulus condition (fear-sensitivity) or between the Face and the Non-Face stimulus condition (face-sensitivity) and the factor Location indicated the EEG channel cluster (Face- vs. Fear-sensitive cluster).

The consistency of the face- and the fear-sensitivity in N290 amplitude across 5 and 7 months was analyzed using Spearman correlation coefficients. To this end, the face-related ERP negativity and fear-related ERP positivity were calculated (from both visits) as difference between the Face and the Non-Face condition, and between the Fear and the Non-Fear condition, respectively.

#### 8.3. Relationships between behavioral and ERP measures

The relationship between the behavioral attention biases and the sensitivity of the N290 amplitude to faces or fearful faces were studied as correlations between: 1) the attention biases and the co-registered N290 response, 2) attention biases at 5 months and the N290 response at 7 months, and 3) N290 response at 5 months and attention biases at 7 months. In these analyses, the attention biases refer to increased attentional dwell on faces or fearful faces (described in ‘8.1 Behavioral analyses’) and the N290 amplitude was expressed as face-related ERP negativity or fear-related ERP positivity (described in ‘8.2 ERP analyses’).

## Results

### 1. Behavioral attention disengagement from faces

An analysis of the current behavioral data has been presented in our previous article [12] indicating an attention bias towards faces and especially to fearful faces. These biases were manifested as an increased probability of an attention dwell for face versus non-face and for fearful as opposed to non-fearful stimuli, respectively. The focus of the current analyses on the attention disengagement data was on the consistency of the attention biases across age as well as on the relationship between the biases and the cortical responses.

The typical behavior to disengage from the face to the peripheral distractor (median probability  = .65 and .56 at 5 and 7 months respectively) was highly consistent across ages (ρ = .40, p<.001, N = 67). In contrast, a low consistence of the behavioral scores was found for both the face- (ρ = −.03, p = .80, N = 67) and the fear-bias (ρ = .09, p<.45, N = 67) across visits. These low correlations may be due to limited reliability of the bias scores (.28 and .39, for the fear- and the face-bias, respectively). Disattenuating the scores [45] indicated that the correlation for the fear-bias across age was .32 in relation to the score reliability. Thus, correlation between the visits may be low because of measurement error.

### 2. Face- and fear-sensitivity in ERPs

**Figure 3:**
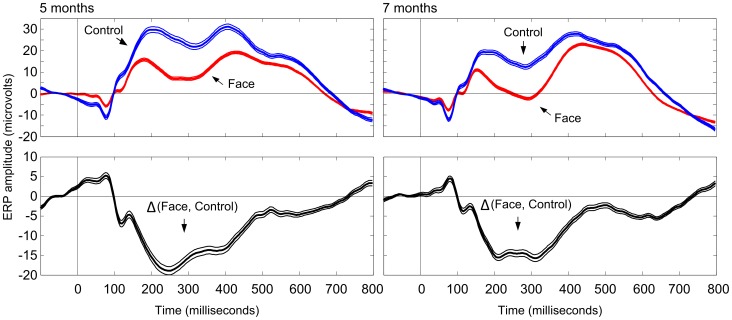
Grand average ERPs from the face- and the non-face conditions extracted from the channel set indicating maximal difference between the conditions. A significant increase in ERP negativity for face vs. non-face stimuli was observed at both 5 and 7 months of age. The effect of face stimulus was indicated over a broad latency range corresponding to the N290 and P400 responses. The confidence intervals were calculated as basic bootstrap intervals comprising 95% of the resampling distribution of mean ERP amplitudes.

**Figure 4:**
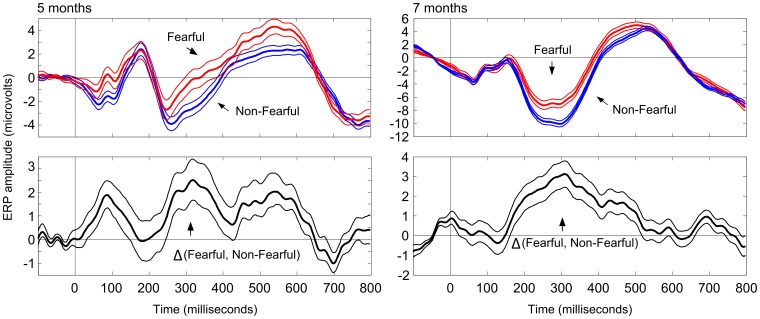
Grand average ERPs from the fear- and the non-fear conditions extracted from the channel set indicating maximal difference between the conditions. A significant increase in ERP positivity for fearful vs. non-fearful stimuli was observed at both 5 and 7 months of age. The effect of stimulus fearfulness was indicated over a broad latency range corresponding to the N290 and P400 responses. The confidence intervals were calculated as basic bootstrap intervals comprising 95% of the resampling distribution of mean ERP amplitudes.

The results of analyses examining the scalp distribution of the face- and fear-effects in the 248–348-ms latency range are shown in Figure 2. N290 waves were elicited by Face and Non-Face (Fig. 3) as well as by fearful and non-fearful stimuli (Fig. 4). The largest differences between the Face- and the Non-Face condition (Figure 2, right) were found in the occipital electrodes (E70, E73, E74, E75, E81, E82, E83, and E88) at both ages. Analyses of differences between the Fear and the Non-Fear condition showed no clear clusters of fear-sensitive electrodes over posterior regions at 5 months, but at 7 months, a positivity for fearful vs. non-fearful faces was observed in medial channels located towards the parietal region (E72, E62, E67, E61, E54, E77, E78, and E79), and coincided with a polarity reversal (negativity) at more anterior sites.

Group-level ERP extraction with bootstrapping and including all available data showed that the face- and fear-effect on ERP amplitudes at the selected electrode clusters were significant at both ages (p<.05), and most evident at the latency of the N290 and early parts of the P400 component (Figures 3 and 4). Additional analyses were also conducted to examine whether the observed effects were dependent on the data analyses techniques used in the current study (particularly detrending) and trial counts. The additional analyses are reported in the Supplementary material (Supplement S1).

To assess the differences in scalp topography between the face- and the fear-sensitive activity in the N290 latency range, the face- and the fear-effect were investigated through Sensitivity × Location interactions on ERP amplitudes. As indicated by Figure 2, the face- and fear-specific modulations in the ERPs were associated with increased scalp negativity and positivity, respectively. This difference in ERP polarity between the face- and fear-sensitivity was reflected in the main effect of Sensitivity at both 5 months [F(1,53) = 34.25, p<.001] and 7 months [F(1,91) = 40.36, p<.001]. The Sensitivity further interacted with Location at both ages [5 months: F(1,53) = 49.00, p<.001; 7 months: F(1,91) = 60.38, p<.001] due to increased face-related negativity at the posterior as opposed to the more anterior electrode cluster (Δ = 14.0 µV and Δ = 13.7 µV at 5 and 7 months, respectively). Thus, the results suggest that the effects of face and fear on cortical activity are dissociable at the level of scalp topography.

In order to track the consistency of face- and fear-sensitivity in N290 amplitude across 5 and 7 months, Spearman correlations between these sensitivities were calculated across visits. The face-sensitivity (increased negativity in Face vs. Control condition) was consistent across 5 and 7 months (ρ = .31, p<.01, N = 50). While fear-related ERP positivity was uncorrelated between visits (ρ = .05, p = .34, N = 63), participants that expressed (above median values of) fear-sensitivity in ERPs at 5 months continued to show higher levels of fear-sensitivity (Fear: M = −5.8 µV, SD = 1.7 µV; Non-Fear: M = −10.7 µV, SD = 1.9) at 7 months of age [F(1,30) = 8.89, p<0.01, Partial *η* ^2^ = 0.23]. Participants with no/low fear-sensitivity in the 5-months ERP data, responded invariantly to fearful (M = −8.5 µV, SD = 2.0 µV) and non-fearful (M = −8.9 µV, SD = 1.2 µV) stimuli [F(1,31) = 0.03, p = 0.86, Partial *η* ^2^ = 0.001].

### 3. Relationships between attention biases and the N290 amplitude

**Figure 5:**
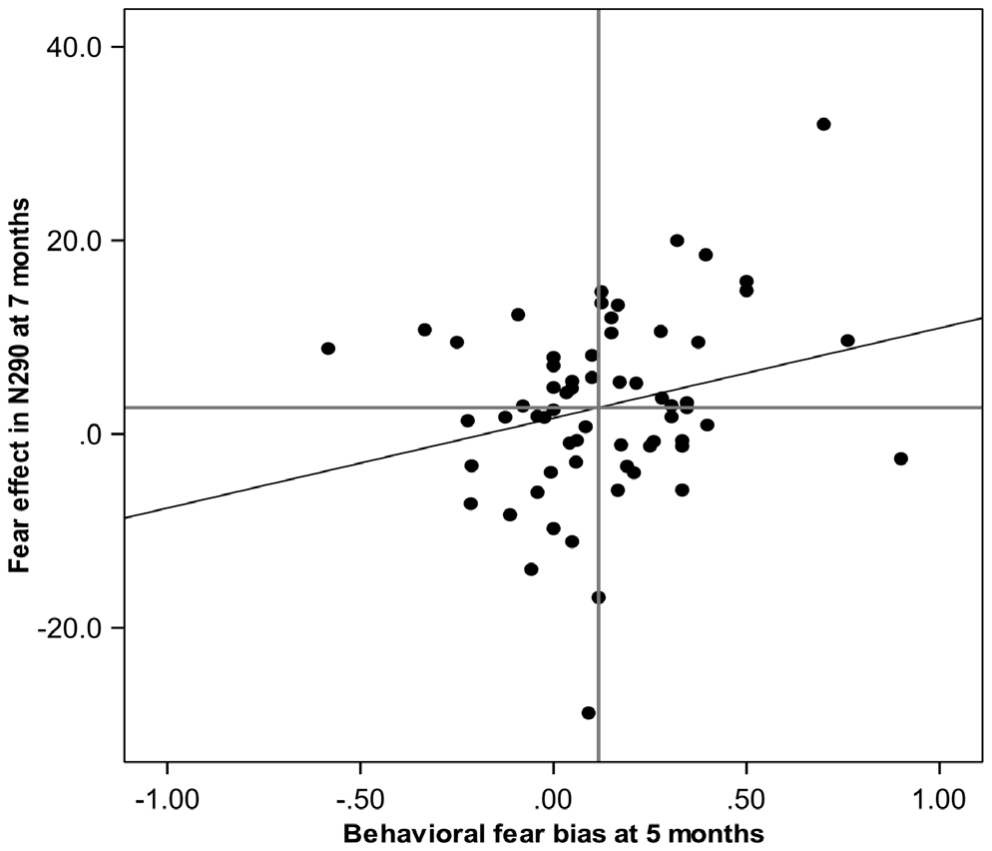
Association between the behavioral fear-bias at 5 months and the ERP fear-sensitivity at 7 months. A positive correlation between the behavioral fear-bias (increased probability of attentional dwell on fearful faces) and fear-sensitivity in N290 amplitude (increased positivity to fearful faces) was found (ρ = .22, p<.05, N = 61). Horizontal and vertical reference lines indicate median values.

No associations between the face- or the fear-bias in attention disengagement and the co-registered N290 amplitude (face- or fear-effect on ERP) were observed at 5 months (|ρs| = .05 to .17, ps = .13 to .36) or at 7 months (|ρs| = .10 to .16, ps = .08 to .24). However, the behavioral fear-bias score calculated from the gaze data from the 5-months measurement was associated with the fear-sensitivity in ERPs at 7 months (ρ = .22, p(1-tailed)<.05, N = 61). That is, an increased behavioral fear-bias at 5 months predicted increased fear-sensitivity in ERPs at 7 months. The mean increase in ERP positivity in the N290 latency range was 4.7 µV (SD = 1.6 µV) and −0.8 µV (SD = 1.6 µV) in participants scoring higher and lower than the median fear-bias, respectively. The relationship between behavioral fear-bias at 5 months and the fear-sensitivity in N290 response at 7 months is illustrated in Figure 5. Note that while the fear-bias correlated with the ERP fear-modulation at 7 months, the absolute proportion of disengagements from face stimuli did not (ρ = −.04 and −.07 for raw disengagement probability at 5 and 7 months, respectively). The face-sensitivity in ERPs at 7 months was uncorrelated with the behavioral bias scores at 5 months (|ρs| = .04 to .08, ps = .26 to .38). Finally, analyses testing the opposite direction of influence (i.e., cortical face/fear-sensitivity as a predictor of later attention bias) showed no correlations between the face-related negativity or fear-related positivity in the N290 at 5 months and the behavioral attention biases at 7 months (|ρs| = .02 to .14., ps = .17 to .46).

## Discussion

By the second half of the first year, infants typically acquire a preference for fearful over other facial expressions [10], [11]. In the current longitudinal study, we tracked this developmental transition and its relationship to the development of cortical face-processing areas from 5 to 7 months of age. The behavioral tendency to suppress attention disengagements from faces as opposed to non-face stimuli and from fearful as opposed to non-fearful stimuli was found at both ages (reported previously in [12]). In the ERP data, facial stimuli with variable emotional expressions elicited face-sensitive cortical activity at both ages as indicated by posterior ERP negativity in the N290 latency range for face as opposed to non-face stimuli. Modulation of cortical activity within the same latency range by stimulus fearfulness was found already at the age of 5 months in the bootstrap analysis including all trials, but this effect became more consistent across different analyses and its scalp distribution more well-defined at 7 months of age. The participants indicating cortical sensitivity to fear at 5 months indicated the fear-effect also at 7 months, expressing the stability of the fear-sensitivity in the ERPs of individual babies. Finally, we analyzed the correlations between attention and ERPs both within and across measurement ages. Our analyses point to the independence of the face- and fear-effects between attention and co-registered face-related cortical activity. However, an interaction between the modalities was revealed as the early fear-bias in attention at 5 months predicted the effect of fear on the follow-up ERP measures of cortical face-specific activity at 7 months.

Unlike in many previous studies examining infants' face and emotion processing, we used a longitudinal design in order to track the development of attention bias towards specific facial expressions at the level of individual babies. While the tendency to disengage from faces per se was highly stable across age, the correlations in the bias scores across the ages of 5 and 7 months turned out to be relatively low. Thus, besides increasing with age in the group level [36], the fear-bias may undergo considerable rank-order changes (for mean-level vs. rank-order changes cf., [46] and [47]) across time in the individual level. The lack of correlation may also be partly explained by attenuation due to measurement error. Our reliability analyses using a split-half design within measurements found low, albeit significant, correlations between scores from the first and the second half of a session. The reliabilities for fear- and face-bias (r = .28 and r = .39, respectively), were representative of those describing behavioral scores previously obtained from infants at this age [48]. Given that our bias scores were summarized from disengagement probabilities from distinct stimulus conditions, their reliability is reduced in comparison to that of the constituent disengagement probabilities. Then again, there were both theoretical and empirical reasons to focus on biases in the attention disengagement rather than on the raw disengagement probabilities. As evidenced by our previous studies, infants typically disengage from the attended stimuli when a distractor is presented. At the same time, this behavior is significantly modulated by the emotive or social value of the attended stimulus [36]. Further, infants' genotype and early life experiences (i.e., parental stress) have been found to be associated with the fear-bias score rather than with raw disengagement probability [12], [42]. Also in our current results, an association between ERPs and behavior was found in the attention preference for fear but not in the general tendency to shift attention from faces to novel objects. Thus, the predictive validity of the attention shift paradigm may be increased by extracting biases for specific emotional signals. However, an important pursuit for future studies would be to increase the reliability of the index of attentional bias. This might be achieved either with protocols that tap more directly to preference between distinct facial stimuli using simultaneous presentation. The reliability of the index may be also increased with improved parameterization of the disengagement data.

The onset of face-sensitive ERP activity at 3 months of age [15], [23] appears to precede sensitivity to fearful faces at around 6 months [24]. It is of note, however, that the relations between cortical face- and fear-sensitive regions have been relatively little investigated in the same sample of infants. The current results replicated previous findings showing face-sensitive activity in the N290 latency range [15]. The scalp distribution of the difference between face- and non-face conditions was remarkably similar between 5 and 7 months of age. The results were more complex regarding fear-sensitivity in ERPs. At 5 months, evidence for fear-sensitivity was observed in the bootstrap analyses only. At 7 months, fear-sensitivity was observed in all analyses, and the scalp distribution of the effect was well-defined and circumscribed to upper posterior electrodes. Together, these results suggest that the expected pattern of posterior positivity for fear may start to emerge at 5 months and become more well-defined (and consistent) at 7 months of age. Finally, the current results indicating distinct ERP scalp topography between the fear- and face-sensitivity together with the known developmental lag between face- and emotion-sensitive ERP effects may reflect modular processing of structural and emotive facial cues during infancy. However, further studies using source level analyses are needed to address the whether these processes truly reflect the activation of separable cortical networks.

While four stimulus categories were presented equiprobably to participants, the analyses of face-sensitivity in the N290 were in effect based on a comparison between ERPs elicited by frequently presented face stimuli (75% incidence) and infrequently presented control stimuli (25% incidence). In this respect, the control stimuli were presented as oddball-deviants which typically elicit ERPs with an increased amplitude [49], [50]. In adults, the visual oddball effect seems to be manifested in early posterior negative wave in the 140–180-ms range [51], [52], which overlaps in time with the face-sensitive N170. While the oddball effect has been established in infants in the auditory domain [53], the effect is susceptible to developmental changes: instead of the early vertex-negative peak elicited by the deviant, a positive wave at 300–400-ms latency range has been observed in 6-months old infants [53], [54]. In infant visual ERPs the amplitude modulation related to oddball stimulation is typically observed in the negative central (Nc) component [55]–[57] which is elicited 350–600 ms after stimulus onset [58], [59], or in the infant P3 [60]. Therefore, the oddball effect in infant visual ERPs seems to have a longer latency than the face-sensitive N290 suggesting that the oddball effect may not play a significant role in the current results. This interpretation is supported by the similarity of the current face-sensitive ERP modulation to that observed in a previous study [15] using an equal probability of face and control stimuli.

Roughly parallel development in behavioral and ERP measures of face processing has been indicated by previous research carried out in infants around 6 months of age. That is, the sensitivity to facial expressions in both behavior and cortical activity seem to emerge during the same period of time. However, longitudinal investigations combining both EEG and behavioral analyses of face-processing are needed to establish the relationships between these measures. On the one hand, the current results indicated a parallelism between the effect of stimulus fearfulness on the ERPs and on the attention allocation on a group level. On the other hand, the within-subjects analyses revealed a more complex relationship between the two phenomena. While the modulations of attention and that of the co-registered ERPs by stimulus fearfulness were largely independent both at 5 and 7 months of age, the attention bias towards fearful faces at 5 months predicted the modulation of the fear-sensitive cortical activity (N290) at 7 months of age. Thus, the gaze preferences and ERP data elicited in the disengagement paradigm seem to reflect two distinct mechanisms which may interact in the development of face processing during infancy.

In interpreting the interaction between attentional bias and emerging cortical sensitivity to facial expressions, it is interesting to note that there is a striking difference between the low maturity of cortical visual areas and the remarkable ability to attend to faces more or less from birth [3]. The early attentional biases related to conspecific detection are argued to be based on a subcortical pathway involving the superior colliculus, pulvinar, and amygdala. Besides developmental precedency of the subcortical over the geniculo-cortical route of face processing, the subcortical pathway modulates cortical processing of faces in adults [61], [62]. It has been further suggested that activation of the amygdala is associated with the allocation of attention towards sensory stimuli in adults [63] and the processing of especially fearful faces [64]–[69]. Importantly, it has been hypothesized that the activity of the subcortical path during face exposure may influence the development of face-processing circuitry in infancy [3]. From these premises, the involvement of the subcortical route of face processing in the currently observed attention bias towards faces and particularly to fearful faces seems possible. The relationship between the fear-bias in attention at 5 months and the fear-sensitivity in ERPs at 7 months could, thus, be tentatively understood as reflecting subcortically driven plasticity in the cortical face-processing areas. Indeed, a potentially critical role of early attention to facial cues in the maturation of social brain networks has previously been indicated by a decline in eye fixation from 2 to 6 months of age in infants later diagnosed with autism [13].

In summary, the current study indicated sensitivity to fearful faces in both cortical activity and behavioral attention biases at ages of 5 and 7 months. The attention bias for fear was related to the development of fear-sensitivity in cortical face-sensitive populations from 5 to 7 months. We suggest that these results may reflect a sensitive period for the development of the cortical encoding of facial cues. In future, it will be important to extend the analyses of behavioral and cortical indices of emotion processing to other components of the infant ERP. For example, a co-analysis of infants' behavioral attentional biases and the Negative central (Nc) component (a long-latency negative component over frontocentral sites) may provide important insights into how infants' attention biases are instantiated on the cortical level as previous studies have shown larger Nc amplitude for fearful as opposed to neutral and happy faces in 6–7 months old infants [24], [25], and the Nc has been interpreted as a neural correlate of attention allocation [70].

## Supporting Information

Supplement S1 Preliminary and complementary ERP analyses. Description of ERP analyses using alternative preprocessing settings and complementary statistical analyses.
(DOC)

Video S1 Illustration of the effects of stimulus condition (Fear vs. Non-Fear) and that of the number of averaged epochs on bootstrapped difference ERPs across series of bootstrap tests.
(AVI)
