# Supplementary material for: Developmental Precursors of Social Brain Networks: The Emergence of Attentional and Cortical Sensitivity to Facial Expressions in 5 to 7 Months Old Infants
Source: PLoS One. 2014 Jun 26;9(6):e100811. doi: 10.1371/journal.pone.0100811 (PMC4072691; doi:10.1371/journal.pone.0100811)
Supplement: Supplement S1 — Preliminary and complementary ERP analyses. Description of ERP analyses using alternative preprocessing settings and complementary statistical analyses. (DOC) [file pone.0100811.s001.doc]

**Supplementary analyses - Developmental precursors of social brain networks, Yrttiaho et al.**

**Preliminary analyses of ERPs to facial expressions**

The analyses included all participants with ≥5 trials per condition, and data from electrodes over occipital temporal regions were averaged by using the same channel groupings as those used in a prior study [1] with a similar, 128-channel electrode layout (Left: 64, 58, 59, 65, 66, 70; Medial; 71, 72, 75, 76; Right; 83, 84, 90, 91, 95, 96). Separate 3 (Channel Group) × 4 (Stimulus Condition) repeated measures analyses of variance were conducted for data from the 5-month (N = 46, Mean 8.5 epochs/condition) and 7-month (N = 78, Mean 8.6 epochs/condition) assessments.

For the 5-month data, there was a significant main effect of Stimulus Condition on the mean ERP amplitude at the latency of the N290 component (248–348 ms), *F*(3, 135) = 10.2, *p* < .001, *partial* *η*^2^ = .19, and a trend-level effect at the latency of the P400 component (348–596 ms), *F*(3, 135) = 2.2, *p* = .088, *partial* *η*^2^ = .05. Planned contrasts examining face- and fear-sensitivity showed differential mean amplitude for non-face control vs. face conditions (i.e., increased negativity for faces) at the latency of the N290 (consistent with [2]), and the P400 component, *F*s (1, 77) > 5.8, *p*s ≤ .05 (Suppl. Figure 1). There were no differences between facial expressions in either of the two latency windows, all *p*s > .10.

For the 7-month data, there were significant effects of Stimulus Condition on the mean ERP amplitude at the latency of the N290 component, *F*(3, 231) = 75.3, *p* < .001, *partial* *η*^2^ = .29, and at that of the P400 component, *F*(3, 231) = 9.4, *p* < .001, *partial* *η*^2^ = .11. Planned contrasts showed differential N290 and P400 amplitude for non-face control vs. face condition, *F*s (1, 77) > 11.1, *p*s ≤ .001 (Suppl. Figure 1). Differential amplitude for non-fearful (i.e., neutral and happy) vs. fearful expressions were also observed, with fearful expressions eliciting larger positivity at the latency of the N290 and P400 components, *F*s (1, 77) > 11.9, *p*s ≤ .001. These results replicate previous studies showing larger posterior positivity for signals of fear in 7-month-old infants with approximately similar time-course [3], [4], although in a prior study with a smaller sample, the positivity for fearful expressions reached statistical significance at latency of the P400 component only [3].

**
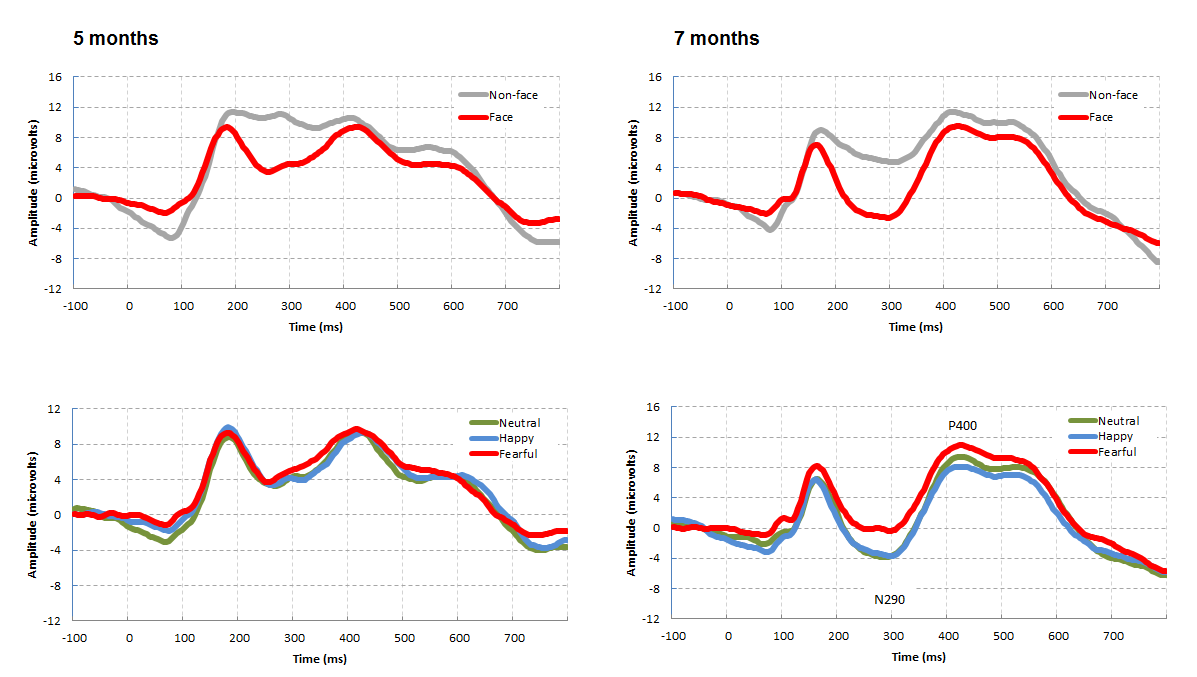
**

*Supplementary Figure 1*. ERPs to non-face control stimuli vs. faces (upper) and faces displaying neutral, happy, and fearful expressions (lower) at 5 and 7 months of age. The ERPs were averaged across all posterior channels included in the analysis.

**Effect of the number of epochs on ERP differences**

The signal to noise ratio of ERP recordings can be increased by augmenting the number of averaged epochs included in the ERP. As the number of epochs was variable across the current experimental conditions, we analyzed whether the observed ERP differences between conditions (e.g., fearful vs. non-fearful) could be due this variability. To this end, a series of bootstrapped difference ERPs were calculated to illustrate the fear-effect (i.e., difference between the fearful and the non-fearful stimulus condition) as well as the effect of epoch count on ERPs. The data from the 5-months visit was selected for these analyses because the issue of epoch count seems more critical here than in the 7-months-visit data where more epochs were available (N = 1726 and N = 862, from the non-fearful and fearful conditions, respectively). The effect of the number of epochs was calculated as a difference between two sets of epochs from the same (non-fearful) condition corresponding either to the full number of available epochs (N = 1266) or to a limited number of epochs (N = 646) matching the number of epochs from the fearful condition.

The bootstrapped ERPs with 95 % confidence intervals (using 100 bootsamples for each ERP), were evaluated 30 times for both the fear-effect and the effect of epoch count. The epochs available for the reduced sample size were selected randomly for each of these ERPs. The ERPs are illustrated in the supplementary video file (Video S1). While the ERPs representing the effect of epoch count occasionally reached non-zero values, the effect of stimulus fearfulness remained consistently larger than these randomly appearing differences. Thus, the effect of stimulus fearfulness reflects a true difference in cortical activity between stimulus conditions and cannot be explained by differences in the number of averaged epochs.

**Effects of detrending**

MATLAB detrend function was applied on a trial-by-trial basis in the primary analyses (presented in the main article text) to eliminate slow drifts from the infant ERP data [5]. Because detrending may introduce biases to the primary data (particularly when the trends are not independent of the experimental manipulations of interest), an additional analysis was conducted to examine whether the primary effect of interest were affected by this preprocessing technique. Analyses examining whether the face-effect was affected by detrending were reported in a previous study [5], and the results of these analyses showed no differences in the time course or magnitude of the face-effect in the detrended vs. not detrended data. The present supplementary analyses examined whether the fear-effect in 7-month-old infants was affected by the employed preprocessing stream. The same processing stream as that used in the primary analyses was used with the exception that some analyses were performed both with and without detrending. The results of the re-analyses, including the scalp topography and time-course of the fear-effect in the original channel set are shown in Supplementary Figure 2. The results of these analyses showed that the posterior positivity for fear is reasonably similar in terms of its scalp topography (Suppl. Fig 2 A) and remarkably similar in time course (Suppl. Fig 2 B-C) in the analyses with *vs.* without detrending. It is noteworthy that the results were not identical, which is likely to result from the fact that substantially larger sample of participants was retained in the analyses with detrending (N = 101) as compared to the analyses without detrending (N = 74).


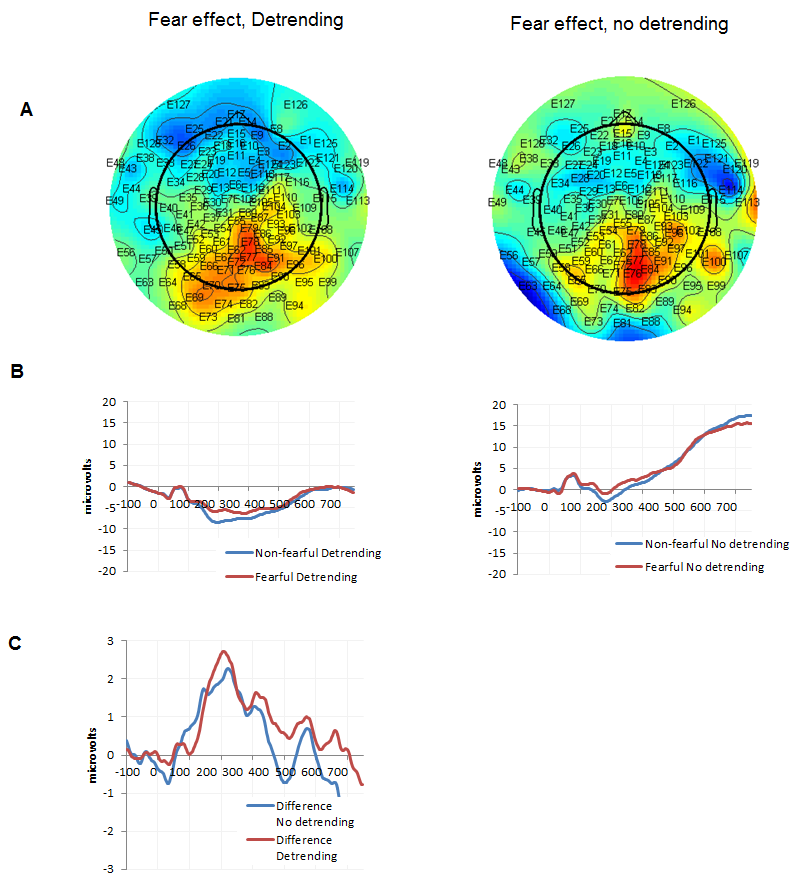


*Supplementary Figure 2***.** Supplementary data showing the scalp topography (A) and time-course (B-C) of the fear-effect in the 7-month data analyzed with and without trial-by-trial detrending.

**Effects of trial number**

A lowered criterion for the number of accepted trials per condition (i.e., ≥ 5) was used in the primary analyses to avoid data loss. This decision was made on the basis of our previous analyses showing that signal preprocessing (detrending) helps in extracting ERPs from a low number of trials, although it does not completely eliminate the effect of trial count on the accuracy of ERP analysis [5]. Thus, re-analyses of the data with differential criteria (trial count ≥ 5, 8, 10) were conducted to examine whether the fear-effect in 7-month-old infants was dependent on trial count. The results of the analysis with de-trending and without de-trending are shown in Supplementary Tables 1 and 2. The results showed that the scalp topography, time-course, and statistical significance of the fear-effect were largely similar across analyses using different criteria.

*Supplementary Table 1*. Sample size (N), statistical significance, scalp topography, and time-course of the fear-effect in analyses using different cut-offs for the number of trials per condition. The analyses were conducted with detrending.

|  | ≥5 trials | ≥8 trials | ≥10 trials |
| --- | --- | --- | --- |
| N^a^ | 101 | 59 | 29 |
| Z^b^ | 2.63 | 3.03 | 2.95 |
| p-value | .008 | .002 | .003 |
| Δ (F, NF)^c^ | 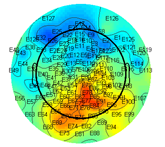 | 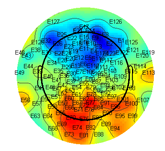 | 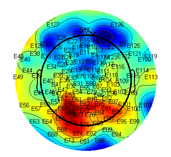 |
| Amplitude  (microvolts)  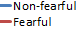 | 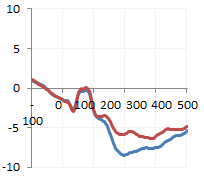 | 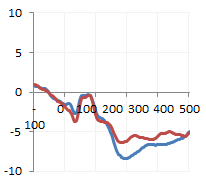 | 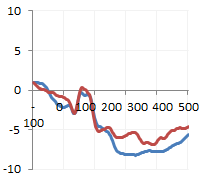 |

^a^Number of participants retained in the analyses

^b^Wilcoxon signed ranks test

^c^F is Fearful, and NF is Non-fearful condition

*Supplementary Table 2*. Sample size (N), statistical significance, scalp topography, and time-course of the fear effect in analyses using different cut-offs for the number of trials per condition. The analyses were conducted without detrending.

| N^a^ | 74 | 33 | 10 |
| --- | --- | --- | --- |
| Z^b^ | 1.9 | 2.14 | 2.09 |
| p-value | .057 | .033 | .037 |
| Δ (F, NF) ^c^ | 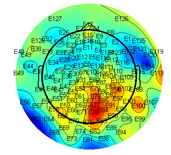 | 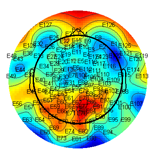 | 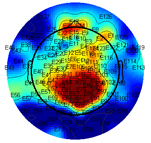 |
| Amplitude  (microvolts)  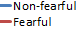 | 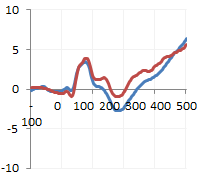 | 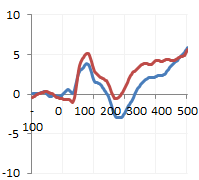 | 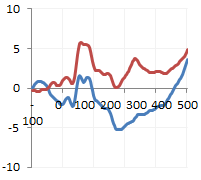 |

^a^Number of participants retained in the analyses

^b^Wilcoxon signed ranks test

^c^F is Fearful, and NF is Non-fearful condition

References

1. Rigato S, Farroni T, Johnson MH (2010) The shared signal hypothesis and neural responses to expressions and gaze in infants and adults. Soc Cogn Affect Neurosci 5: 88–97. doi:10.1093/scan/nsp037.
2. Halit H, Csibra G, Volein Á, Johnson MH (2004) Face-sensitive cortical processing in early infancy. J Child Psychol Psychiatry 45: 1228–1234.
3. Leppänen JM, Moulson MC, Vogel-Farley VK, Nelson CA (2007) An ERP study of emotional face processing in the adult and infant brain. Child Dev 78: 232–245.
4. Leventon JS, Bauer PJ (2013) The sustained effect of emotional signals on neural processing in 12-month-olds. Dev Sci 16: 485–498. doi:10.1111/desc.12041.
5. Kaatiala J, Yrttiaho J, Forssman L, Leppänen J (2013) A graphical user interface for infant ERP analysis. Behav Res Methods. In press. doi: 10.3758/S13428-013-0404-4.
